# Genome-wide detection of genetic markers associated with growth and fatness in four pig populations using four approaches

**DOI:** 10.1186/s12711-017-0295-4

**Published:** 2017-02-14

**Authors:** Yuanmei Guo, Yixuan Huang, Lijuan Hou, Junwu Ma, Congying Chen, Huashui Ai, Lusheng Huang, Jun Ren

**Affiliations:** State Key Laboratory of Pig Genetic Improvement and Production Technology, Jiangxi Agricultural University, Nanchang, 330045 China

## Abstract

**Background:**

Genome-wide association studies (GWAS) have been extensively used to identify genomic regions associated with a variety of phenotypic traits in pigs. Until now, most GWAS have explored single-trait association models. Here, we conducted both single- and multi-trait GWAS and a meta-analysis for nine fatness and growth traits on 2004 pigs from four diverse populations, including a White Duroc × Erhualian F_2_ intercross population and Chinese Sutai, Laiwu and Erhualian populations.

**Results:**

We identified 44 chromosomal regions that were associated with the nine traits, including four genome-wide significant single nucleotide polymorphisms (SNPs) on SSC2 (SSC for *Sus scrofa* chromosome), 4, 7 and X. Compared to the single-population GWAS, the meta-analysis was less powerful for the identification of SNPs with population-specific effects but more powerful for the detection of SNPs with population-shared effects. Multiple-trait analysis reduced the power to detect trait-specific SNPs but significantly enhanced the power to identify common SNPs across traits. The SNP on SSC7 had pleiotropic effects on the nine traits in the F_2_ and Erhualian populations. Another pleiotropic SNP was observed on SSCX for these traits in the F_2_ and Sutai populations. Both population-specific and shared SNPs were identified in this study, thus reflecting the complex genetic architecture of pig growth and fatness traits.

**Conclusions:**

We demonstrate that the multi-trait method and the meta-analysis on multiple populations can be used to increase the power of GWAS. The two significant SNPs on SSC7 and X had pleiotropic effects in the F_2_, Erhualian and Sutai populations.

**Electronic supplementary material:**

The online version of this article (doi:10.1186/s12711-017-0295-4) contains supplementary material, which is available to authorized users.

## Background

Growth and fatness traits are economically important and have been intensively selected in the global pig industry. Dissection of the genetic architecture of growth and fat deposition in pigs not only benefits the pig industry but also sheds insight into our understanding of human obesity, because pigs are more physiologically similar to humans than rodents and other model animals [1].

To understand the molecular basis of divergent phenotypes in pigs, researchers have established multiple F_2_ intercross populations using European and Chinese breeds as founders, and have mapped quantitative trait loci (QTL) for a list of phenotypic traits, including growth and fatness traits, using hundreds of microsatellite markers across the whole genome [2–6]. Until now, 1880 and 1070 QTL for growth and fatness traits have been deposited in the pig QTL database (http://www.animalgenome.org/cgi-bin/QTLdb/SS/index, Release 26, Apr 27, 2015), respectively. These findings have significantly advanced our understanding of the genetic architecture of porcine growth and fatness traits. Nevertheless, the resolution of traditional QTL mapping is relatively poor due to markers being sparse and insufficient recombination events in the F_2_ crosses. Thus, identification of causative mutations that underlie the identified QTL remains a big challenge.

With the availability of the Illumina Porcine SNP60 Beadchip, it has become feasible to exploit the association between high-density single nucleotide polymorphisms (SNPs) and phenotypic traits through genome-wide association studies (GWAS) [7]. Compared to the traditional QTL mapping approach, the GWAS approach allows the identification of SNPs that are significantly associated with traits. Nevertheless, large sample sizes are still required to identify SNPs that are weakly associated with target traits. A meta-analysis of GWAS can not only increase statistical power but also reduce the number of false positives by combining information from multiple independent studies [8]. Moreover, for a QTL with pleiotropic effects on multiple traits, a multi-trait analysis can improve the detection power of GWAS [9–11].

The aim of our study was to identify SNPs associated with nine traits related to growth and fatness across four pig populations, including a White Duroc × Erhualian F_2_ intercross (referred hereafter as F_2_), Sutai, Laiwu and Erhualian pigs by four GWAS methods: single-trait analysis on a single population (SS-GWAS), single-trait analysis on multiple populations (SM-GWAS), multi-trait analysis on a single population (MS-GWAS), and multi-trait analysis on multiple populations (MM-GWAS).

## Methods

### Ethics statement

All procedures used for this study and involving animals are in compliance with guidelines for the care and utility of experimental animals established by the Ministry of Agriculture of China. The ethics committee of Jiangxi Agricultural University specifically approved this study.

#### Animals and phenotypic measurements

A total of 2004 pigs were used in this study, including 925, 434, 331 and 314 individuals from the F_2_, Sutai, Erhualian and Laiwu populations, respectively. The F_2_ and Sutai populations were previously described in [12, 13]. Briefly, the F_2_ animals originated from a cross between two White Duroc boars and 17 Erhualian sows [12]. In this population, nine F_1_ boars and 59 F_1_ sows were intercrossed by avoiding full-sib mating to produce 1912 F_2_ pigs. A total of 925 F_2_ pigs were randomly selected from all F_1_ boar and sow families. These animals were slaughtered at the age of 240 ± 3 days and carcass and meat quality traits were measured; the remaining F_2_ pigs were used to produce F_3_ individuals or measure the reproductive traits. The Sutai pig is a Chinese synthetic breed that was derived from a cross between Western Duroc boars and Chinese Taihu (mainly Erhualian) sows. This breed has experienced directional selection for prolificacy and growth for more than 18 generations. The 434 Sutai pigs used in the current study were offspring of four sires and 55 dams [13]. Erhualian and Laiwu are Chinese indigenous breeds. The former is known for its prolificacy, with a litter size that can exceed 15, and the latter is characterized by its exceptionally high intramuscular fat content (more than 9%) [14]. We obtained 334 Erhualian (168 sires and 166 dams) and 314 Laiwu (218 sires and 98 dams) pigs at the age of ~90 days from two national conservation farms of the two breeds in Jiangsu and Shandong provinces, respectively. The F_2_, Sutai, Erhualian and Laiwu pigs were all raised in an experimental farm in Nanchang, Jiangxi province from 2001 to 2014. All 2004 pigs had ad libitum access to fresh water and consistent feed containing 16% crude protein, 3100 kJ of digestive energy, and 0.78% lysine during the fattening period. Each pig in the four populations was weighed at birth and at 210 and 240 days of age, and the average daily gains from 0 to 210 days of age (ADG_0–210_) and from 210 to 240 days of age (ADG_210–240_) were calculated. The F_2_ and Sutai pigs were slaughtered in the same commercial abattoir at 240 ± 3 days of age and the Erhualian and Laiwu pigs at 300 ± 3 days of age. After slaughter, all pigs were measured for fatness traits, including backfat thickness at the shoulder (SBF), the first rib (FBF), the last rib (LBF), and at the hip (HBF), and weight of leaf fat (LFW), veil fat (VFW) and abdominal fat (AFW).

### Genotyping and quality control

Genomic DNA was extracted from ear or tail tissues using a standard phenol/chloroform protocol, and was then quantified and adjusted to a final concentration of 50 ng/µl. All 2004 pigs were genotyped with the porcine 60 K SNP Beadchip on an iScan System (Illumina, USA) following the manufacturer’s protocol. SNPs, including sex-linked SNPs, that had a call rate less than 95%, a minor allele frequency lower than 5%, or that strongly deviated from Hardy–Weinberg equilibrium (*P* < 0.000001) were discarded. Animals with a call rate less than 95% were also removed from further analyses. These quality controls were performed for each population separately to include as many qualified SNPs as possible for the GWAS in each population. A common set of 15,429 qualified SNPs across the four populations was used in the meta-analysis of GWAS.

### Statistical methods

Descriptive statistics of phenotypic traits were calculated by the MEANS procedure of SAS9.0 (SAS Institute Inc., USA) and phenotypic differences between sexes were tested by the TTEST procedure. The MIXED procedure was used to determine the fixed effects and the covariates included in the GWAS model. Sex and fattening batch were included as fixed effects in all GWAS models, and birth weight, body weight at 210 days, and carcass weight were treated as covariates for ADG_0–210_, ADG_210–240_ and for fatness traits, respectively, in the GWAS models. A polygenic effect for each animal with covariances based on genomic kinship, which was calculated based on the identity-by-state of the SNPs on autosomes [15, 16], was included as a random effect to account for the effect of population substructure. The *P* values of Bonferroni corrected thresholds for suggestive, 5 and 1% genome-wide significant levels were 1, 0.05 and 0.01, respectively, divided by the number of SNPs used in the GWAS. The suggestive level was first proposed by Lander and Kruglyak [17] and represents the threshold where, under the null hypothesis, one false positive is expected per genome scan.

#### GWAS for a single trait in a single population (SS-GWAS)

The two-stage approach implemented in the R package GenABEL was used to conduct SS-GWAS under an additive model. First, the following mixed model was used to calculate the phenotypic residual vector ${\mathbf{e}}^{*}$:

$${\mathbf{y}} = {\mathbf{Xb}} + {\mathbf{Zu}} + {\mathbf{e}}^{*} ,$$

where ${\mathbf{y}}$ is a vector of phenotypes; ${\mathbf{b}}$ is the estimator vector of fixed effects, including population mean *μ*; ${\mathbf{u}}$ is a vector of random polygenic additive effects that follows a normal distribution $N\left( { {\mathbf{0}} , {\mathbf{G}}\upsigma_{\text{u}}^{2} } \right)$, with ${\mathbf{G}}$ being the genomic kinship matrix calculated from all autosomal SNPs based on identity-by-state [16], $\upsigma_{\text{u}}^{2}$ is the polygenic additive variance, and ${\mathbf{X}}$ and ${\mathbf{Z}}$ are the incidence matrices for ${\mathbf{b}}$ and ${\mathbf{u}}$, respectively.

Then, a family-based score test was used to detect associations between SNPs and traits using the following simple regression model [18], one SNP at a time:

$${\mathbf{e}}^{*} = {\mathbf{S}}{\text{a}} + {\mathbf{e}},$$

where ${\text{a}}$ is an estimator of the SNP allele substitution effect; ${\mathbf{S}}$ is the incidence vector for ${\text{a}}$ (coded 0, 1, 2 based on allele dosage); and ${\mathbf{e}}$ is a vector of residual errors that follows a normal distribution $N\left( { {\mathbf{0}} , {\mathbf{I}}\upsigma_{\text{e}}^{2} } \right)$, with ${\mathbf{I}}$ being an identity matrix and $\upsigma_{\text{e}}^{2}$ the variance of the residual error. The *P* value of the association test was adjusted by the genomic control method to correct for residual inflation [19, 20].

#### GWAS for multiple traits in a single population (MS-GWAS)

MS-GWAS were conducted to detect pleiotropic SNPs using the method proposed by Bolormaa et al. [11]. In brief, a Chi square statistic, which approximately follows a Chi square distribution with the number of traits tested as the number of degrees of freedom, was calculated for each SNP using the following formula:

$$\chi _{{multi-trait}}^{2} = {\mathbf{t}}_{i}^{\prime } {\mathbf{V}}^{{ - 1}} {\mathbf{t}}_{i} ,$$

where ${\mathbf{t}}_{i}$ is a 9 × 1 vector of the signed $t$-values for the $i$th SNP from the SS-GWAS for the nine traits, ${\mathbf{t}}_{i}^{\prime }$ is the transpose of ${\mathbf{t}}_{i}$, and ${\mathbf{V}}^{ - 1}$ is the inverse of the 9 × 9 correlation matrix between traits. The correlation between two traits was calculated by correlating the estimated effects (signed $t$-values) of the 15,429 qualified SNPs for the two traits.

#### Meta-analysis of GWAS for a single trait (SM-GWAS) and multiple traits (MM-GWAS)

We applied the inverse variance weighting method of [8], in which each population is weighted according to the inverse of its squared standard error, to perform SM-GWAS and MM-GWAS across the four populations based on the results of SS-GWAS and MS-GWAS, respectively. The weight ($w_{i}$) for the $i$th population was equal to the inverse of the square of the standard error ($s_{i}$) of the allele substitution effect in the $i$th population. Then, the pooled estimates of the allele effect ($\beta$) of a given SNP and its standard error ($s$) were calculated as follows:

$$\beta = \frac{{\mathop \sum \nolimits_{i = 1}^{n} w_{i} \beta_{i} }}{{\mathop \sum \nolimits_{i = 1}^{n} w_{i} }}\;{\text{and}}\;s^{2} = \frac{1}{{\mathop \sum \nolimits_{i = 1}^{n} w_{i} }},$$

where $n$ is the population number; $\beta_{i}$ is the allele effect in the $i$th population.

A statistic ($Z$ score) of *Z*-test was calculated as follows:

$$Z = \frac{\beta }{s} = \frac{{\mathop \sum \nolimits_{i = 1}^{n} w_{i} \beta_{i} }}{{\sqrt {\mathop \sum \nolimits_{i = 1}^{n} w_{i} } }}.$$

An allele of an associated SNP may have a positive effect in some populations and a negative effect in the others. Such an inconsistent effect could significantly reduce the detection power of the meta-analysis of GWAS. To circumvent this, we used information on linkage disequilibrium and ignored information on phase, i.e., the absolute value of $\beta_{i}$ was used to calculate the pooled $\beta$ and $Z$ values.

To determine if a SNP that was significantly associated with multiple traits was due to closely linked genes or pleiotropy, we conducted a conditional SS-GWAS by fixing the effect of the top SNPs identified by MS-GWAS in the statistic model.

#### Linkage and linkage disequilibrium analyses

To determine the approximate genomic positions of the unmapped significant SNPs, a two-point linkage analysis was used to detect linkage between the mapped and unmapped SNPs in the F_2_ population [21]. The lower the recombination rate ($\theta$) is, the tighter is the link between the two SNPs. Haplotypes of the regions that were significantly associated with the target traits were inferred by Simwalk2.9 [22], and linkage disequilibrium blocks were defined using default parameters of Haploview4.2 [23]. The VennDiagram in R package was used to draw a Venn diagram that showed the loci that were in common among the four populations and the four methods.

## Results

### Descriptive statistics of the traits

The descriptive statistics for the nine growth and fatness traits in the F_2_ and Sutai populations were reported in our previous publications [24, 25]. Additional file 1: Table S1 shows the means and standard errors for these nine traits as well as the phenotypic differences between females and males in the Erhualian and Laiwu populations. Growth rates were not significantly different between sexes in the two populations, except that Erhualian males grew faster (*P* = 0.03) than females from 210 to 240 days of age. Backfat thickness at the four localizations was significantly higher (*P* < 10^−6^) in males than in females in the Erhualian population, while no significant difference was observed in the Laiwu population. Males deposited more (*P* < 0.01) leaf fat, while females stored more (*P* < 10^−10^) fat in the abdomen in both populations. Veil fat was significantly heavier (*P* = 6.77 × 10^−6^) in males than in females in Erhualian pigs, while there was no significant difference in veil fat between sexes in Laiwu pigs.

### Phenotypic correlation coefficients between traits

Raw phenotypic correlation coefficients between the measured traits in the two populations are in Additional file 2: Table S2. All correlation coefficients between the measured traits were significant (*P* < 0.05) in the Erhualian and Laiwu populations, except that between ADG_0–210_ and ADG_210–240_ in the Laiwu population. In general, fatness traits were more significantly correlated with each other than growth traits in both populations. ADG_0–210_ had a moderate positive correlation with the other traits and ADG_210–240_ showed a weak positive correlation with the other traits.

### Qualified SNPs and animals in the GWAS

All genotyped animals passed quality control with SNP call rates higher than 0.95. A total of 34,636 (4825), 36,341 (4979), 24,602 (3492) and 32,058 (4527) mapped (unmapped) SNPs were qualified for GWAS in the F_2_, Sutai, Erhualian and Laiwu populations, respectively. The *P* values of the 5% (suggestive) genome-wide significant threshold were equal to 1.27 × 10^−6^ (2.53 × 10^−5^), 1.21 × 10^−6^ (2.42 × 10^−5^), 1.78 × 10^−6^ (3.56 × 10^−5^) and 1.37 × 10^−6^ (2.73 × 10^−5^) in these four populations, respectively. Based on the pig genome assembly (Sscrofa10.2, http://www.ensembl.org/Sus_scrofa/Info/Index), the average physical distances between adjacent SNPs were 74.7, 71.2, 105.1 and 80.7 kb in these four populations, respectively. A common set of 15,429 qualified SNPs across the four populations was used in the GWAS meta-analysis, with average physical distance between adjacent SNPs of 167.7 kb. The *P* values of the 5% genome-wide and suggestive significant thresholds were equal to 3.24 × 10^−6^ and 6.48 × 10^−5^, respectively, in the meta-analysis.

### SNPs identified by single-trait GWAS

**Fig. 1:**
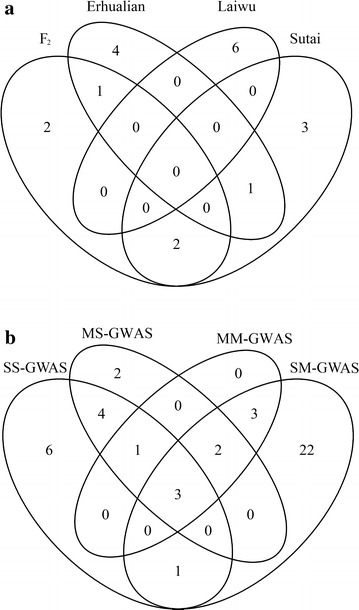
Venn diagram showing common loci identified in this study. **a** Common loci between four populations. **b** Common loci between four methods: SS-GWAS, single-trait GWAS in single population; SM-GWAS, single-trait GWAS in multiple populations (meta-analysis); MS-GWAS, multi-trait GWAS in single population; MM-GWAS, multi-trait GWAS in multiple populations (meta-analysis)

Table 1 shows the genome-wide significant regions for the nine fatness and growth traits identified by the four GWAS approaches in this study. The SS-GWAS and SM-GWAS identified 15 and 31 chromosomal regions (loci) associated with these nine traits (Table 1; Fig. 1 [see Additional file 3: Table S3, Additional file 4: Figure S1]), respectively. Four loci were consistently detected by the two methods, one each on SSC1, 2, 7 and X.

**Table 1:** Genome-wide significant loci for nine fatness and growth traits identified by four GWAS approaches in this study

QTL^a^	Chr^b^	Trait^c^	Population	Method	Top SNP	Pos Mb^d^	Effect ± SE^e^	*P* value^f^	*N*_SNP_^g^	Boundary SNPs	
1	2	HBF	Meta	SM	ss131211507	3.61	0.127 ± 0.026	1.28E−06*	1	–	–
2	4	Multi-trait	Meta	MM	ss131269439	81.71	–	7.88E−10**	8	ss131269439	ss478935222
2	4	FBF	Meta	SM	ss131269439	81.71	0.168 ± 0.032	1.20E−07**	1	–	–
2	4	HBF	Meta	SM	ss131269678	82.25	0.156 ± 0.032	1.08E−06*	1	–	–
2	4	SBF	Meta	SM	ss478935222	85.09	0.166 ± 0.035	1.53E−06**	1	–	–
3	6	ADG_210–240_	Meta	SM	ss131029816	71.60	0.040 ± 0.008	1.38E−06*	1	–	–
4	7	ADG_210_	F_2_	SS	ss131342496	32.96	-0.026 ± 0.005	5.95E−07*	1	–	–
4	7	Multi-trait	Meta	MM	ss131343534	34.56	–	2.65E−18**	27	ss120018804	ss131348342
4	7	SBF	Meta	SM	ss131343534	34.56	0.255 ± 0.042	1.11E−09**	6	ss131341589	ss107890951
4	7	FBF	Meta	SM	ss131343534	34.56	0.315 ± 0.040	4.88E−15**	17	ss131341589	ss131348342
4	7	HBF	Meta	SM	ss131343534	34.56	0.379 ± 0.045	3.16E−17**	25	ss120018804	ss131348342
4	7	Multi-trait	F_2_	MS	ss107837325	34.80	–	3.97E−33**	107	ss23131766	ss478941636
4	7	AFW	F_2_	SS	ss107837325	34.80	0.163 ± 0.022	8.99E−11**	34	ss131066868	ss131345041
4	7	FBF	F_2_	SS	ss107837325	34.80	0.691 ± 0.060	3.10E−17**	69	ss131341676	ss131348342
4	7	HBF	F_2_	SS	ss107837325	34.80	0.773 ± 0.072	1.97E−14**	64	ss131341676	ss131347489
4	7	LBF	F_2_	SS	ss107837325	34.80	0.571 ± 0.057	1.77E−14**	60	ss131341676	ss131348342
4	7	LFW	F_2_	SS	ss107837325	34.80	0.650 ± 0.058	1.75E−18**	63	ss131341766	ss131347489
4	7	SBF	F_2_	SS	ss107837325	34.80	0.487 ± 0.057	1.94E−12**	45	ss131341766	ss131346335
4	7	Multi-trait	Erhualian	MS	ss131343870	34.84	–	1.29E−15**	6	ss131342502	ss131347459
4	7	FBF	Erhualian	SS	ss131343870	34.84	0.585 ± 0.073	2.13E−13**	8	ss131336720	ss131347459
4	7	HBF	Erhualian	SS	ss131343870	34.84	0.502 ± 0.075	1.49E−10**	5	ss131343870	ss131347459
4	7	LBF	Erhualian	SS	ss131343870	34.84	0.523 ± 0.064	6.96E−14**	5	ss131343870	ss131347459
4	7	LFW	Erhualian	SS	ss131343870	34.84	0.453 ± 0.067	2.39E−10**	5	ss131343870	ss131347459
4	7	SBF	Erhualian	SS	ss131343870	34.84	0.453 ± 0.079	4.08E−08**	2	ss131343870	ss478941599
4	7	LFW	Meta	SM	ss131344553	36.20	0.140 ± 0.028	7.18E−07*	2	ss131344553	ss131347175
4	7	LBF	Meta	SM	ss131347175	40.85	0.155 ± 0.024	1.27E−10**	13	ss131337529	ss131348342
5	X	SBF	Meta	SM	ss131570179	46.75	0.124 ± 0.020	5.42E−10**	5	ss131036304	ss131562987
5	X	VFW	F_2_	SS	ss107834496	51.70	0.091 ± 0.013	5.39E−10**	21	ss478944418	ss478935791
5	X	Multi-trait	F_2_	MS	ss23131102	63.65	–	5.78E−35**	79	ss131067158	ss131563360
5	X	FBF	F_2_	SS	ss23131102	63.65	0.164 ± 0.023	2.94E−07*	2	ss478943984	ss23131102
5	X	HBF	F_2_	SS	ss23131102	63.65	0.278 ± 0.029	6.81E−12**	24	ss478936157	ss131562911
5	X	Multi-trait	Sutai	MS	ss478934917	78.58	–	3.19E−10**	38	ss478944000	ss131570171
5	X	Multi-trait	Meta	MM	ss131070541	106.48	–	1.61E−15**	14	ss478936157	ss131562987
5	X	FBF	Meta	SM	ss131070541	106.48	0.110 ± 0.018	1.33E−09**	10	ss131036304	ss131562987
5	X	HBF	Meta	SM	ss131070541	106.48	0.187 ± 0.021	9.06E−19**	18	ss131067158	ss131563051
5	X	LBF	Meta	SM	ss131070541	106.48	0.108 ± 0.018	1.46E−09**	1	–	–

^a^We operationally define two loci with the distance between their lead SNPs less than 5 Mb as the same QTL except the loci on chromosome X. All loci close to the recombination cold spot (more than 30 Mb, with an extremely low rate of recombination) in the middle of X chromosome was considered the same QTL because of too few SNPs in this region

^b^Chromosome

^c^Abbreviations of trait names are in Additional file 1: Table S1

^d^The position of the unmapped SNP (ss131029816) is deduced by its tightly linked SNP (ss131566312)

^e^The direction of SNP effect estimated by the meta-GWAS was not shown because the linkage phase may be inconsistent among the four populations, and the SNP effect cannot be estimated by the multi-trait GWAS

^f^** 1% genome-wide significant; * 5% genome-wide significant

^g^Number of SNPs that surpass the significance level

#### SS-GWAS

In the F_2_ population, the most promising locus was mapped at 34.8 Mb on SSC7 (Table 1). Another genome-wide significant locus was identified in the middle of chromosome X. Two suggestive loci were detected, one on SSC1 and one on SSC3 [see Additional file 3: Table S3]. Five and four suggestive loci were identified in the Sutai and Laiwu datasets, respectively. No locus was significant at the genome-wide level in these two datasets. The loci on SSC1, 2 and 8 were detected exclusively for the Sutai population [see Additional file 3: Table S3, Additional file 4: Figure S1]. A genome-wide locus was identified at 34.84 Mb on SSC7 for the Erhualian population. Another three suggestive loci were identified, including two population-specific loci, one each on SSC4 and 6 [see Additional file 3: Table S3, Additional file 4: Figure S1].

#### SM-GWAS

The SM-GWAS identified 31 loci that were distributed on all chromosomes except SSC11 and 13, including 22 loci that were not identified in the SS-GWAS and four genome-wide loci, one each on SSC2, 4, 7 and X, that were also identified in the SS-GWAS (Fig. 1; Table 2, and Table S3 [see Additional file 3: Table S3]). These four genome-wide loci were all associated with backfat thickness at the four evaluated sites, and the loci on SSC4 and 7 were also associated with ADG and LFW, respectively.

**Table 2:** Genome-wide significant SNPs detected by the meta-analysis of single-trait GWAS common to the four populations, with corresponding additive effects

Chr^a^	Trait^b^	Top SNP	Pos Mb	Allele	Effect ± SE^c^			
					F_2_	Sutai	Erhualian	Laiwu
2	HBF	ss131211507	3.61	A	0.149 ± 0.046**	0.146 ± 0.039**	0.051 ± 0.066	−0.042 ± 0.121
4	FBF	ss131269439	81.71	A	−0.148 ± 0.061*	−0.114 ± 0.060	−0.234 ± 0.068**	−0.198 ± 0.067**
4	HBF	ss131269678	82.25	A	−0.292 ± 0.076**	0.110 ± 0.046*	0.048 ± 0.077	−0.256 ± 0.079**
4	SBF	ss478935222	85.09	G	−0.176 ± 0.063**	−0.129 ± 0.050*	0.323 ± 0.136*	−0.196 ± 0.087*
7	SBF	ss131343534	34.56	G	0.420 ± 0.058**	0.094 ± 0.088	−0.083 ± 0.094	0.020 ± 0.169
7	FBF	ss131343534	34.56	G	0.565 ± 0.062**	0.097 ± 0.078	−0.194 ± 0.088*	−0.082 ± 0.125
7	HBF	ss131343534	34.56	G	0.655 ± 0.074**	0.266 ± 0.085**	−0.225 ± 0.089*	0.047 ± 0.145
7	LFW	ss131344553	36.20	G	0.325 ± 0.070**	0.119 ± 0.033**	0.296 ± 0.092**	0.146 ± 0.070*
7	LBF	ss131347175	40.85	A	−0.435 ± 0.062**	−0.050 ± 0.033	−0.315 ± 0.068**	0.119 ± 0.054*
X	SBF	ss131570179	46.75	A	−0.162 ± 0.030**	−0.084 ± 0.030**	−0.127 ± 0.072	−0.137 ± 0.088
X	HBF	ss131070541	106.48	G	−0.234 ± 0.026**	−0.028 ± 0.054	−0.145 ± 0.056**	−0.179 ± 0.080**
X	LBF	ss131070541	106.48	G	−0.141 ± 0.023**	0.029 ± 0.045	−0.058 ± 0.048	−0.091 ± 0.064
X	FBF	ss131070541	106.48	G	−0.131 ± 0.022**	−0.051 ± 0.052	−0.057 ± 0.055	−0.079 ± 0.070

^a^Chromosome

^b^Abbreviations of trait names are in Additional file 1: Table S1

^c^The significant level is a single point without Bonferroni correction: ** *P* ≤ 0.01; * *P* ≤ 0.05

### Loci identified by multi-trait GWAS

#### MS-GWAS

**Fig. 2:**
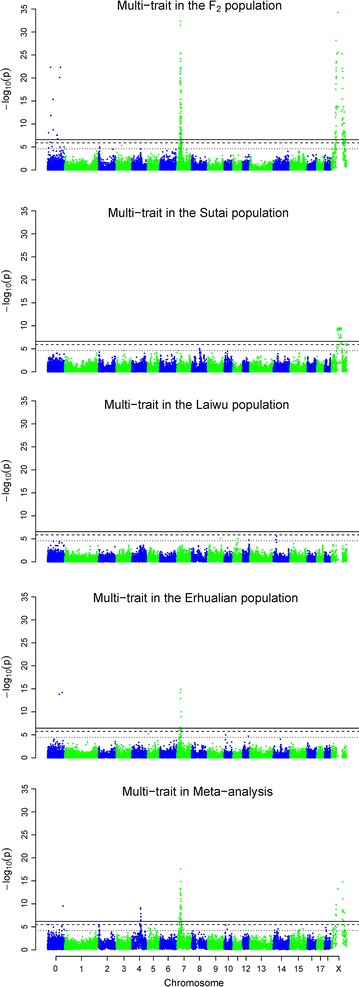
Manhattan plots of the multi-trait GWAS and meta-analysis for fatness and growth traits in F_2_, Sutai, Laiwu and Erhualian populations. The *solid*, *dashed* and *dotted horizontal lines* indicate the 1 and 5% genome-wide and suggestive significant threshold values, respectively. Unmapped SNPs are assigned on chromosome 0 and arbitrary ordered by their names

The MS-GWAS revealed 12 loci associated with both fatness and growth traits, including two loci with genome-wide significance (Fig. 2; Table 1; Additional file 3: Table S3). Four loci were identified for the F_2_ population, including two genome-wide significant loci, one at 34.80 Mb on SSC7 and one at 63.65 Mb on chromosome X. For the Sutai population, three loci were identified, including one genome-wide locus at 78.58 Mb on SSCX. For the Laiwu population, only four suggestive loci were identified. Four loci were detected for the Erhualian population, including one genome-wide locus at 34.84 Mb on SSC7 (Fig. 2).

#### MM-GWAS

Nine loci were identified in the MM-GWAS, including three genome-wide significant loci on SSC4, 7 and X (Fig. 2). All nine loci were identified by the SM-GWAS, except the suggestive locus at 10.53 Mb on SSC5 (Fig. 1).

### Additive effects of top SNPs in the four populations

The additive effects of top SNPs at genome-wide and suggestive significant levels for the four populations are in Table 2 and see Additional file 5: Table S4, respectively. Thirteen SNPs were associated with fatness traits at the genome-wide significant level in the SM-GWAS. Among these 13 SNPs, five had a consistent direction of additive effects across the four populations, while five showed inconsistent effect directions. The other three SNPs just achieved the significance level for one population. We observed a similar pattern for suggestive SNPs: 12 consistent SNPs and nine inconsistent SNPs. This indicates that the linkage phases between these SNPs and the causative mutations that underlie the detected QTL are not always identical across populations.

## Results of the conditional GWAS

**Fig. 3:**
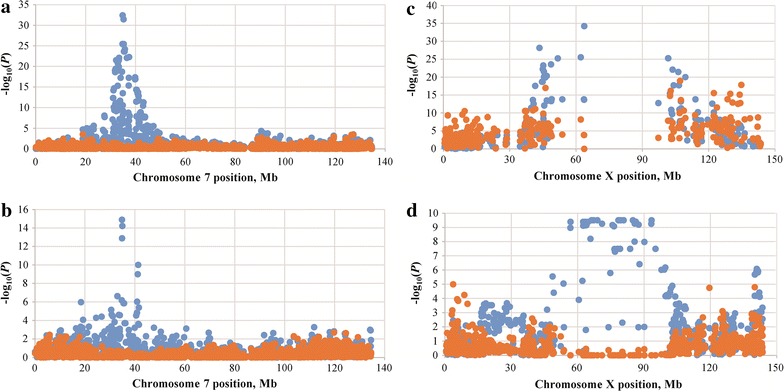
Log_10_(*P*) of the multi-trait test before (*blue*) and after (*orange*) fitting the top SNPs at the loci with genome-wide significance. **a** Chromosome 7 in the F_2_ population. **b** Chromosome 7 in the Erhualian population. **c** Chromosome X in the F_2_ population. **d** Chromosome X in the Sutai population

We identified two loci associated with multiple traits at the genome-wide significant level by the MS-GWAS. One region was located on SSC7 (between 34.80 and 34.84 Mb) for the F_2_ and Erhualian populations, and the other mapped to SSCX (between 63.65 and 66.25 Mb) for the F_2_ and Sutai populations. After fixing the effects of the top SNPs (detected by the MS-GWAS) in the SS-GWAS model, we found that no SNP on SSC7 showed any association signal with multiple traits for both the F_2_ and Erhualian populations (Fig. 3a, b). No SNP was significantly associated with the nine traits in the region between 60 and 90 Mb on SSCX for the F_2_ and Sutai populations, respectively (Fig. 3c, d).

## Discussion

### Comparison of our results with previously reported loci

Previously, we had identified 15 significant loci on 11 chromosomes for the traits measured in the F_2_ and Sutai populations that were also analysed here [25]. In the current study, we identified nine loci on six chromosomes for the same traits by single-trait and multi-trait GWAS in these two populations. Only four loci (one each on SSC2, 4, 7 and X, see Tables 1, 2) were common between both studies and have also been consistently reported in previous studies [2–4, 6, 24, 25]. The lower power in the current study can be attributed to the fact that residual inflation was not corrected in our previous study, but was corrected by genomic control here. To date, 1880 QTL for growth and 1070 for fatness traits have been reported in the pig QTL database [26]. Although we conducted GWAS on four divergent populations using four approaches, no novel loci were identified in the current study. The four GWAS methods yielded different results with few common results, which is likely due to the fact that each method has its own advantage to identify distinct loci. For example, the multi-trait analysis is suitable for detecting pleiotropic loci, the meta-analysis is sufficiently powerful to identify common loci across populations, and the single-population GWAS is an effective method to detect population-specific loci.

### Unmapped SNPs improve the power of association analyses

As shown in our previous studies [13, 27, 28], retaining the unmapped SNPs can improve the power of GWAS. In this study, we identified 18 unmapped SNPs that surpassed the suggestive or genome-wide significant level [see Additional file 4: Figure S1]. By applying linkage analysis in the F_2_ population, we deduced the approximate genomic locations of 15 unmapped SNPs [see Additional file 6: Table S5], and the use of these SNPs improved the power of the current GWAS. For example, we did not identify any mapped SNPs associated with ADG_210–240_ in the Sutai dataset. However, a SNP (ss131031851) that had two locations (65.29 and 65.38 Mb) on SSC10 displayed an association signal with ADG_210–240_ [see Additional file 4: Figure S1, Additional file 6: Table S5]. The unmapped SNP ss131029816 is another example. We detected only one mapped SNP on SSC6 that was associated with ADG_210–240_ at a suggestive significant level in the SM-GWAS, while the unmapped SNP ss131029816 showed a genome-wide significant association with this trait [see Additional file 4: Figure S1, Additional file 6: Table S5]. In a two-point linkage analysis on the F_2_ population, ss131029816 was tightly linked (*θ* = 0.0022) to ss131566312 at 71.6 Mb on SSC6. Therefore, a genome-wide significant locus for ADG_210–240_ was identified at around 71.6 Mb on SSC6.

### Single-population GWAS versus meta-analysis

The SS-GWAS showed that four loci were common to two populations (Fig. 1a). Two loci, one at the proximal end of SSC2 and one in the middle of SSCX, were shared between the F_2_ and Sutai populations (Table 1 and Table S3 [see Additional file 3: Table S3]). The locus around 34.80 Mb on SSC7 was associated with fatness traits in both the F_2_ and Erhualian datasets (Table 1). This locus may be segregating in the population of Laiwu pigs since we observed a weak signal around it (Fig. 1; see Additional file 4: Figure S1). Another locus in the middle of SSC12 was found to be shared between the Erhualian and Laiwu populations. The segregation of these common loci in multiple populations should allow us to efficiently fine map these loci by using higher-density chips, such as the 600 K SNP chip, since inter-population linkage disequilibrium usually extends over short distances ($r_{0.3}^{2} = \, 10.5$ kb) in Chinese pigs [29].

We noted that there were no common loci across three or four populations in the SS-GWAS. However, the SM-GWAS identified 31 significant loci that were putative common loci across these four populations (Fig. 1b). One explanation for the discrepancy between the two GWAS methods is that the detection power of SS-GWAS is lower than that of SM-GWAS because of the small sizes of individual populations, which prevents the detection of these putative common loci. Another possibility is that some informative SNPs in the SS-GWAS were deleted during the filtering process in the SM-GWAS. Of the 31 loci, 13 surpassed the genome-wide significant level. It is interesting that all 13 SNPs had ‘single point significant’ effects in the F_2_ population, while only some of these SNPs showed such effects in the other three populations (Table 2). This is most likely due to the fact that the F_2_ population has the largest population size and thus had greater impact on the results of the meta-analysis.

**Fig. 4:**
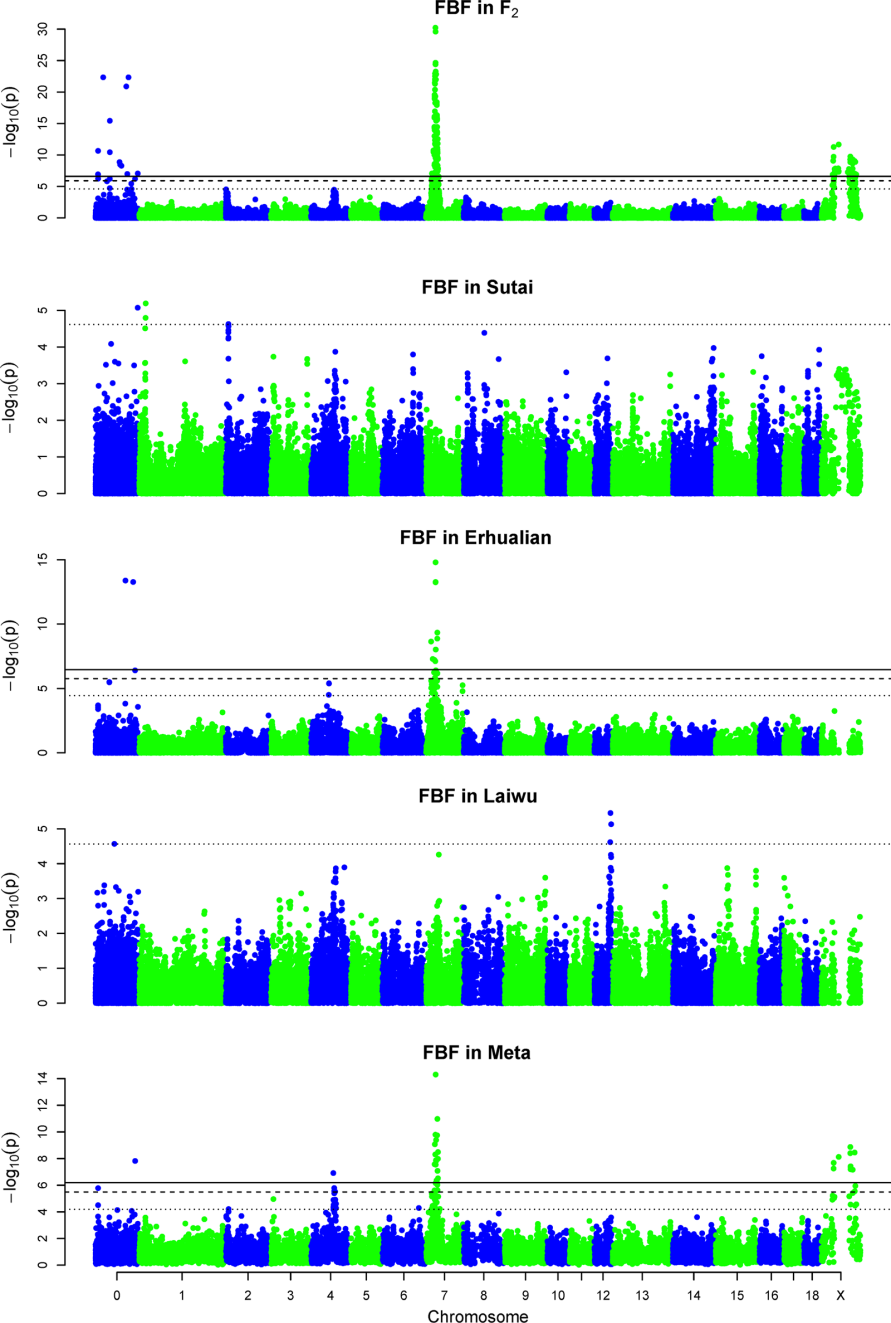
Manhattan plots of the single-trait GWAS and meta-analysis for backfat thickness at first rib (FBF) in F_2_, Sutai, Laiwu and Erhualian populations. The *solid*, *dashed* and *dotted horizontal lines* indicate the 1 and 5% genome-wide and suggestive significant threshold values, respectively. Unmapped SNPs are assigned on chromosome 0 and arbitrary ordered by their names

It is known that meta-analysis of GWAS can improve the detection power when two or more populations show consistent association signals with target traits. For example, we detected a suggestive locus for FBF at 65.72 Mb on SSC4 in the Erhualian population [see Additional file 3: Table S3], while this region had only a weak (non significant) association signal with FBF in the other three populations (Fig. 4). By performing a meta-analysis across the four populations, we observed an association signal at the 1% genome-wide significant level in this region (Fig. 4; Table 1). However, population-specific loci could not be detected by the meta-analysis. For instance, we identified an F_2_-specific locus for ADG_210–240_ at 53.06 Mb on SSC3. This locus was not detected by the meta-analysis.

### Single-trait GWAS versus multi-trait GWAS

If a locus has pleiotropic effects on multiple traits, the multi-trait method can be used to enhance the power of GWAS. For example, the SS-GWAS identified 15 loci in this study, more than half of which were also identified by the MS-GWAS (Fig. 1b). In the SS-GWAS, the minimum *P* values at the locus on SSC7 for the F_2_ and Erhualian populations were 1.75E−18 and 6.96E−14, respectively; whereas these two values decreased to 3.97E−33 and 1.29E−15 in the MS-GWAS, respectively. In contrast, if a locus has no pleiotropic effects, the MS-GWAS can decrease the signal. For instance, the suggestive locus at 303.92 Mb on SSC1 for ADG_0–210_ in the F_2_ population was identified by the SS-GWAS but not by the MS-GWAS. Furthermore, it has been reported that multi-trait GWAS can map the locus more accurately than the single-trait method when the marker density is high [11]. It should be noted that the extent of linkage disequilibrium is usually large in F_2_ populations, for which a multi-trait GWAS based on medium-density SNP chips, such as 60 K SNPs, is expected to have high detection power.

### Linkage versus pleiotropy

By applying multi-trait GWAS, we detected three prominent loci on SSC4, 7 and X (Fig. 2), respectively. The detection of these loci could be caused by pleiotropy or closely-linked causal QTL. The SSC7 locus detected for the F_2_ and Erhualian populations, as well as the SSCX locus for the Sutai pigs (Fig. 2), are most likely loci with pleiotropic effects, since we found no SNP that showed an association signal with the nine traits when the top SNPs were fixed in the MS-GWAS model (Fig. 3a, b, d). After correction for the effect of the top SNP on SSCX, several SNPs were still associated with the traits tested in F_2_ pigs (Fig. 3c). This implies that the locus on SSCX could be caused by closely-linked causal variants in F_2_ pigs.

## Conclusions

In this study, we explored four GWAS approaches to identify genomic loci for nine growth and fatness traits in four pig populations. Compared to the single-trait analysis, the meta-analysis had less power to identify population-specific loci but more power to detect population-shared loci. Compared to the single-trait analysis, the multiple-trait analysis reduced the power to detect trait-specific loci but enhanced the power to identify the common loci across traits. Our findings demonstrate that the meta-analysis and the multi-trait method can be used to increase the power of GWAS.

## Additional files

**Additional file 1: Table S1.** Descriptive statistics and differences between females and males for nine growth and fatness traits in the Erhualian and Laiwu pigs.

**Additional file 2: Table S2.** Phenotypic correlation coefficients between nine growth and fatness traits in the Laiwu and Erhualian pigs.

**Additional file 3: Table S3.** The suggestive significant loci for nine fatness and growth traits identified in this study.

**Additional file 4: Fig. S1.** Manhattan plots of the single-trait GWAS and meta-analysis for nine fatness and growth traits in the F_2_, Sutai, Laiwu and Erhualian populations. The solid, dashed and dotted horizontal lines indicate the 1% and 5% genome-wide and suggestive significant threshold values, respectively. Unmapped SNPs are assigned on chromosome 0 and arbitrary ordered by their names.

**Additional file 5: Table S4.** The additive effects of suggestive significant SNPs detected by the meta-analysis in the four populations.

**Additional file 6: Table S5.** Tight linkage of unassigned significant SNPs with the mapped SNPs. This table shows approximate genomic positions of the unassigned significant SNPs by a two-point linkage analysis in the F_2_ population.
