# Supplementary material for: Genome-wide detection of genetic markers associated with growth and fatness in four pig populations using four approaches
Source: Genet Sel Evol. 2017 Feb 14;49:21. doi: 10.1186/s12711-017-0295-4 (PMC5307927; doi:10.1186/s12711-017-0295-4)
Supplement: Supplementary file 1 — Additional file 1: Table S1. Descriptive statistics and differences between females and males for nine growth and fatness traits in the Erhualian and Laiwu pigs. [file 12711_2017_295_MOESM1_ESM.doc]

**Table S1** Descriptive statistics and differences between females and males for nine growth and fatness traits in the Erhualian and Laiwu pigs.

| **Trait** | **Symbol** | **Male** | | **Female** | | **Male-Female** | **All** |
| --- | --- | --- | --- | --- | --- | --- | --- |
|  |  | *N* | Mean±S.E. | *N* | Mean±S.E. | Mean±S.E. | Mean±S.E. |
| **Erhualian** |  |  |  |  |  |  |  |
| ADG from birth to 210 day, kg/day | ADG_0-210_ | 161 | 0.25±0.005 | 161 | 0.24±0.004 | 0.01±0.006 | 0.24±0.003 |
| ADG from 210 to 240 day, kg/day | ADG_210-240_ | 149 | 0.41±0.012 | 156 | 0.37±0.011 | 0.04±0.017^*^ | 0.39±0.008 |
| Abdominal fat weight, kg | AFW | 168 | 0.80±0.021 | 166 | 1.08±0.025 | -0.27±0.033^**^ | 0.94±0.018 |
| Leaf fat weight, kg | LFW | 168 | 3.11±0.075 | 166 | 2.33±0.059 | 0.78±0.095^**^ | 2.72±0.052 |
| Veil fat weight, kg | VFW | 168 | 1.41±0.030 | 166 | 1.21±0.025 | 0.20±0.039^**^ | 1.31±0.020 |
| Backfat thickness at shoulder, cm | SBF | 168 | 4.59±0.076 | 166 | 4.02±0.065 | 0.57±0.100^**^ | 4.31±0.052 |
| Backfat thickness at first rib, cm | FBF | 168 | 4.09±0.071 | 166 | 3.45±0.059 | 0.64±0.092^**^ | 3.77±0.049 |
| Backfat thickness at last rib, cm | LBF | 168 | 2.68±0.056 | 166 | 2.25±0.050 | 0.43±0.075^**^ | 2.46±0.039 |
| Backfat thickness at hip, cm | HBF | 168 | 3.13±0.069 | 166 | 2.64±0.060 | 0.50±0.091^**^ | 2.88±0.047 |
| **Laiwu** |  |  |  |  |  |  |  |
| ADG from birth to 210 day, kg/day | ADG_0-210_ | 187 | 0.27±0.004 | 63 | 0.26±0.007 | 0.01±0.009 | 0.26±0.004 |
| ADG from 210 to 240 day, kg/day | ADG_210-240_ | 201 | 0.48±0.010 | 85 | 0.50±0.015 | -0.02±0.018 | 0.48±0.008 |
| Abdominal fat weight, kg | AFW | 218 | 1.24±0.023 | 98 | 1.57±0.045 | -0.33±0.050^**^ | 1.34±0.023 |
| Leaf fat weight, kg | LFW | 218 | 4.37±0.079 | 98 | 3.97±0.120 | 0.41±0.143^**^ | 4.25±0.067 |
| Veil fat weight, kg | VFW | 218 | 1.50±0.027 | 97 | 1.49±0.044 | 0.01±0.052 | 1.50±0.023 |
| Backfat thickness at shoulder, cm | SBF | 218 | 4.98±0.068 | 98 | 4.90±0.101 | 0.08±0.122 | 4.95±0.056 |
| Backfat thickness at first rib, cm | FBF | 218 | 4.38±0.056 | 98 | 4.35±0.085 | 0.03±0.102 | 4.37±0.047 |
| Backfat thickness at last rib, cm | LBF | 218 | 2.96±0.046 | 98 | 2.97±0.078 | -0.02±0.090 | 2.96±0.040 |
| Backfat thickness at hip, cm | HBF | 218 | 4.05±0.065 | 98 | 4.09±0.095 | -0.04±0.115 | 4.06±0.054 |

* and ** indicate the differences between sexes at 5% and 1% significant levels, respectively.
