# Supplementary material for: Genome-wide detection of genetic markers associated with growth and fatness in four pig populations using four approaches
Source: Genet Sel Evol. 2017 Feb 14;49:21. doi: 10.1186/s12711-017-0295-4 (PMC5307927; doi:10.1186/s12711-017-0295-4)
Supplement: Supplementary file 2 — Additional file 2: Table S2. Phenotypic correlation coefficients between nine growth and fatness traits in the Laiwu and Erhualian pigs. [file 12711_2017_295_MOESM2_ESM.doc]

**Table S2** Phenotypic correlation coefficients between nine growth and fatness traits in the Laiwu and Erhualian pigs

|  | **SBF** | **FBF** | **LBF** | **HBF** | **AFW** | **LFW** | **VFW** | **ADG_0-210_** | **ADG_210-240_** |
| --- | --- | --- | --- | --- | --- | --- | --- | --- | --- |
| **SBF** |  | 0.6491 | 0.4984 | 0.5090 | 0.2982 | 0.4916 | 0.3550 | 0.3820 | 0.1574 |
| **FBF** | 0.7887 |  | 0.7374 | 0.6830 | 0.4283 | 0.5903 | 0.4527 | 0.4161 | 0.1182 |
| **LBF** | 0.7168 | 0.8259 |  | 0.6582 | 0.4413 | 0.5528 | 0.3627 | 0.3733 | 0.1830 |
| **HBF** | 0.7037 | 0.7809 | 0.7475 |  | 0.4222 | 0.6051 | 0.3807 | 0.4372 | 0.1753 |
| **AFW** | 0.3424 | 0.3330 | 0.3004 | 0.3727 |  | 0.4647 | 0.3382 | 0.4460 | 0.2600 |
| **LFW** | 0.6452 | 0.7728 | 0.7134 | 0.7269 | 0.3326 |  | 0.6162 | 0.6206 | 0.2152 |
| **VFW** | 0.6020 | 0.6766 | 0.6063 | 0.6069 | 0.4306 | 0.7896 |  | 0.4250 | 0.2033 |
| **ADG_0-210_** | 0.4441 | 0.4515 | 0.3990 | 0.4975 | 0.4944 | 0.5726 | 0.5667 |  | -0.0047 |
| **ADG_210-240_** | 0.2800 | 0.2943 | 0.2964 | 0.3338 | 0.1884 | 0.3665 |  |  |  |

1. The symbols are defined in Table S1; 2. The correlation coefficients in the Laiwu and Erhualian populations are listed in the upper and lower triangles, respectively; 3. All correlation coefficients are significant except the one between ADG_0-210_ and AGD_210-240_ in the Laiwu population.
