# Supplementary material for: Genome-wide detection of genetic markers associated with growth and fatness in four pig populations using four approaches
Source: Genet Sel Evol. 2017 Feb 14;49:21. doi: 10.1186/s12711-017-0295-4 (PMC5307927; doi:10.1186/s12711-017-0295-4)
Supplement: Supplementary file 3 — Additional file 3: Table S3. The suggestive significant loci for nine fatness and growth traits identified in this study. [file 12711_2017_295_MOESM3_ESM.doc]

**Table S3 The suggestive significant loci for fatness and growth traits identified in this study**

| **QTL No.^1^** | **Chr^2^** | **Trait^3^** | **Population** | **Method** | **Top SNP** | **Position, Mb** | **Effect ± SE^4^** | ***P* value^5^** | ***N*_SNP_^6^** | **Boundary SNPs** | |
| --- | --- | --- | --- | --- | --- | --- | --- | --- | --- | --- | --- |
| 6 | 1 | FBF | Sutai | SS | ss131139967 | 24.03 | 0.257 ± 0.057 | 1.40E-05 | 1 | - | - |
| 7 | 1 | LFW | Laiwu | SS | ss131127953 | 133.33 | 0.740 ± 0.160 | 1.47E-05 | 2 | ss131127896 | ss131127953 |
| 8 | 1 | HBF | Meta | SM | ss107832889 | 279.55 | 0.131 ± 0.032 | 3.34E-05 | 1 | - | - |
| 9 | 1 | VFW | Meta | SM | ss131154610 | 294.77 | 0.062 ± 0.015 | 6.05E-05 | 1 | - | - |
| 9 | 1 | ADG_0-210_ | F_2_ | SS | ss131158638 | 303.92 | 0.018 ± 0.004 | 1.77E-05 | 1 | - | - |
| 1 | 2 | Multi-trait | Meta | MM | ss131211507 | 3.61 | - | 5.51E-06 | 3 | ss131211507 | ss131193699 |
| 1 | 2 | LBF | Meta | SM | ss131211507 | 3.61 | 0.089 ± 0.021 | 2.74E-05 | 1 | - | - |
| 1 | 2 | LFW | Sutai | SS | ss131191281 | 7.66 | 0.235 ± 0.052 | 2.09E-05 | 1 | - | - |
| 1 | 2 | SBF | Meta | SM | ss131193581 | 9.37 | 0.118 ± 0.026 | 4.87E-06 | 1 | - | - |
| 1 | 2 | FBF | Meta | SM | ss131193699 | 9.58 | 0.100 ± 0.025 | 6.07E-05 | 1 | - | - |
| 1 | 2 | Multi-trait | F_2_ | MS | ss107875878 | 11.16 | - | 9.98E-06 | 2 | ss120020385 | ss107875878 |
| 10 | 2 | LFW | Meta | SM | ss131197985 | 117.07 | 0.113 ± 0.027 | 2.70E-05 | 1 | - | - |
| 11 | 2 | AFW | Meta | SM | ss131201823 | 132.20 | 0.043 ± 0.010 | 3.74E-05 | 1 | - | - |
| 12 | 2 | HBF | Sutai | SS | ss131114532 | 150.00 | 0.222 ± 0.045 | 1.42E-05 | 1 | - | - |
| 13 | 3 | FBF | Meta | SM | ss131215218 | 9.48 | 0.101 ± 0.023 | 1.09E-05 | 1 | - | - |
| 13 | 3 | LFW | Meta | SM | ss131054825 | 9.96 | 0.109 ± 0.027 | 3.73E-05 | 1 | - | - |
| 14 | 3 | Multi-trait | F_2_ | MS | ss131215182 | 53.06 | - | 4.80E-06 | 1 | - | - |
| 14 | 3 | ADG_210-240_ | F_2_ | SS | ss131215182 | 53.06 | 0.061 ± 0.014 | 1.40E-05 | 2 | ss131234753 | ss131215182 |
| 15 | 3 | Multi-trait | Sutai | MS | ss131218864 | 69.71 | - | 2.33E-05 | 1 | - | - |
| 16 | 3 | AFW | Meta | SM | ss107873924 | 109.12 | 0.047 ± 0.011 | 2.41E-05 | 1 | - | - |
| 17 | 4 | FBF | Erhualian | SS | ss478940911 | 65.72 | 0.502 ± 0.109 | 2.19E-05 | 1 | - | - |
| 2 | 4 | ADG_0-210_ | Meta | SM | ss131267570 | 77.15 | 0.011 ± 0.003 | 5.84E-05 | 2 | ss131267000 | ss131267570 |
| 2 | 4 | ADG_210-240_ | Meta | SM | ss131269053 | 80.96 | 0.034 ± 0.008 | 1.33E-05 | 6 | ss131267772 | ss131243891 |
| 2 | 4 | LBF | Meta | SM | ss131269678 | 82.25 | 0.110 ± 0.025 | 7.84E-06 | 3 | ss131269678 | ss107807591 |
| 18 | 5 | Multi-trait | Erhualian | MS | ss131116205 | 8.24 | - | 6.03E-06 | 1 | - | - |
| 18 | 5 | ADG_0-210_ | Erhualian | SS | ss131116205 | 8.24 | 0.021 ± 0.005 | 3.00E-05 | 1 | - | - |
| 18 | 5 | Multi-trait | Meta | MM | ss131298766 | 10.53 | - | 1.86E-05 | 4 | ss131298766 | ss131288929 |
| 19 | 5 | LFW | Meta | SM | ss120032250 | 25.79 | 0.117 ± 0.028 | 2.88E-05 | 1 | - | - |
| 19 | 5 | LBF | Meta | SM | ss107903259 | 27.07 | 0.113 ± 0.025 | 4.58E-06 | 3 | ss107903259 | ss131283779 |
| 19 | 5 | ADG_0-210_ | Meta | SM | ss478941239 | 28.66 | 0.011 ± 0.003 | 2.40E-05 | 2 | ss478941239 | ss131297315 |
| 20 | 5 | AFW | Meta | SM | ss120032165 | 87.87 | 0.056 ± 0.014 | 5.31E-05 | 1 | - | - |
| 21 | 6 | ADG_0-210_ | Meta | SM | ss131110597 | 9.27 | 0.010 ± 0.002 | 3.19E-05 | 1 | - | - |
| 22 | 6 | ADG_210-240_ | Meta | SM | ss131094986 | 90.68 | 0.026 ± 0.006 | 4.85E-05 | 1 | - | - |
| 23 | 6 | FBF | Meta | SM | ss107829154 | 134.48 | 0.108 ± 0.027 | 5.05E-05 | 1 | - | - |
| 24 | 6 | ADG_0-210_ | Erhualian | SS | ss131317336 | 157.47 | 0.020 ± 0.005 | 2.02E-05 | 1 | - | - |
| 4 | 7 | VFW | Erhualian | SS | ss131343870 | 34.84 | 0.132 ± 0.028 | 4.63E-06 | 3 | ss131336720 | ss478941599 |
| 4 | 7 | VFW | F_2_ | SS | ss107806758 | 35.18 | 0.132 ± 0.225 | 2.65E-06 | 14 | ss131343748 | ss131345041 |
| 25 | 8 | LBF | Meta | SM | ss107826859 | 15.01 | 0.095 ± 0.023 | 2.65E-05 | 3 | ss107826860 | ss131567211 |
| 26 | 8 | Multi-trait | Sutai | MS | ss131371347 | 76.98 | - | 8.72E-06 | 4 | ss131079769 | ss131103017 |
| 26 | 8 | SBF | Sutai | SS | ss131371347 | 76.98 | 0.447 ± 0.100 | 1.87E-05 | 1 | - | - |
| 26 | 8 | LBF | Sutai | SS | ss131371347 | 76.98 | 0.345 ± 0.074 | 1.78E-05 | 1 | - | - |
| 27 | 8 | AFW | Meta | SM | ss131376236 | 117.96 | 0.042 ± 0.010 | 1.34E-05 | 1 | - | - |
| 28 | 9 | AFW | Meta | SM | ss107867089 | 12.14 | 0.054 ± 0.013 | 2.78E-05 | 3 | ss107867089 | ss131039062 |
| 29 | 9 | LBF | Meta | SM | ss131390688 | 47.49 | 0.095 ± 0.024 | 6.43E-05 | 1 | - | - |
| 30 | 9 | Multi-trait | Laiwu | MS | ss107809334 | 121.97 | - | 8.11E-06 | 1 | - | - |
| 31 | 10 | Multi-trait | Meta | MM | ss131434909 | 12.79 | - | 4.17E-06 | 3 | ss131434489 | ss131032897 |
| 31 | 10 | Multi-trait | Erhualian | MS | ss107814954 | 12.72 | - | 1.07E-05 | 1 | - | - |
| 31 | 10 | LFW | Meta | SM | ss131434909 | 12.79 | 0.098 ± 0.023 | 2.73E-05 | 5 | ss131434188 | ss131434909 |
| 32 | 10 | AFW | Meta | SM | ss131032897 | 27.69 | 0.089 ± 0.022 | 4.46E-05 | 1 | - | - |
| 33 | 10 | VFW | Meta | SM | ss131086122 | 54.23 | 0.053 ± 0.012 | 1.96E-05 | 3 | ss131086122 | ss131028698 |
| 34 | 11 | Multi-trait | Laiwu | MS | ss120017978 | 45.50 | - | 8.98E-06 | 1 | - | - |
| 34 | 11 | SBF | Laiwu | SS | ss120017978 | 45.50 | 0.361 ± 0.079 | 1.02E-05 | 1 | - | - |
| 35 | 12 | ADG_0-210_ | Meta | SM | ss131459427 | 31.23 | 0.013 ± 0.003 | 1.64E-05 | 1 | - | - |
| 35 | 12 | VFW | Meta | SM | ss131073797 | 36.81 | 0.051 ± 0.012 | 1.96E-05 | 1 | - | - |
| 36 | 12 | Multi-trait | Erhualian | MS | ss131126557 | 54.00 | - | 1.96E-05 | 1 | - | - |
| 36 | 12 | Multi-trait | Laiwu | MS | ss107812785 | 58.95 | - | 1.85E-05 | 1 | - | - |
| 36 | 12 | FBF | Laiwu | SS | ss107812785 | 58.95 | 0.262 ± 0.057 | 7.10E-06 | 2 | ss107812785 | ss131109904 |
| 37 | 14 | Multi-trait | Laiwu | MS | ss107812933 | 31.45 | - | 2.66E-06 | 2 | ss131509696 | ss107812933 |
| 37 | 14 | HBF | Meta | SM | ss107868905 | 37.75 | 0.130 ± 0.032 | 5.07E-05 | 1 | - | - |
| 37 | 14 | Multi-trait | Meta | MM | ss131512461 | 45.22 | - | 2.80E-05 | 2 | ss131498214 | ss131512461 |
| 38 | 14 | LFW | Meta | SM | ss131502410 | 137.22 | 0.126 ± 0.031 | 3.78E-05 | 1 | - | - |
| 39 | 15 | LFW | Meta | SM | ss478935207 | 12.81 | 0.119 ± 0.028 | 1.61E-05 | 1 | - | - |
| 39 | 15 | ADG_210-240_ | Meta | SM | ss131531233 | 16.45 | 0.024 ± 0.006 | 5.43E-05 | 1 | - | - |
| 40 | 15 | ADG_210-240_ | Laiwu | SS | ss131523358 | 35.94 | 0.070 ± 0.015 | 5.95E-06 | 2 | ss131523358 | ss131523352 |
| 41 | 15 | Multi-trait | Meta | MM | ss120029026 | 121.31 | - | 2.82E-05 | 3 | ss107897157 | ss120029026 |
| 41 | 15 | SBF | Meta | SM | ss120029026 | 121.31 | 0.137 ± 0.033 | 4.27E-05 | 1 | - | - |
| 42 | 16 | ADG_210-240_ | Meta | SM | ss131536391 | 52.37 | 0.033 ± 0.007 | 1.44E-05 | 1 | - | - |
| 43 | 17 | AFW | Meta | SM | ss131549596 | 5.76 | 0.038 ± 0.009 | 6.41E-05 | 1 | - | - |
| 44 | 17 | VFW | Meta | SM | ss131543346 | 19.96 | 0.046 ± 0.011 | 6.03E-05 | 1 | - | - |
| 45 | 18 | Multi-trait | Meta | MM | ss71868563 | 12.98 | - | 1.41E-05 | 2 | ss71868563 | ss131559348 |
| 45 | 18 | HBF | Meta | SM | ss71868563 | 12.98 | 0.122 ± 0.028 | 1.18E-05 | 1 | - | - |
| 5 | X | ADG_0-210_ | Sutai | SS | ss107852986 | 48.60 | 0.016 ± 0.004 | 1.75E-05 | 1 | - | - |
| 5 | X | HBF | Sutai | SS | ss478934917 | 78.58 | 0.148 ± 0.028 | 2.90E-06 | 42 | ss478944000 | ss478944083 |
| 5 | X | LBF | F_2_ | SS | ss131070541 | 106.48 | 0.141 ± 0.023 | 1.58E-06 | 8 | ss478943984 | ss131563331 |
| 5 | X | SBF | F_2_ | SS | ss131073728 | 115.08 | 0.198 ± 0.034 | 1.33E-06 | 19 | ss478944418 | ss131073728 |

1. The loci within 10 Mb were considered as the same QTL except the loci on chromosome X. Since there is a big region with extremely low rate of recombination in the middle of chromosome X, the loci detected from 46.75 – 115.08 Mb were thought the same locus; 2. Chromosome; 3. The abbreviations of the traits are as same as those in Table S1; 4. The direction of SNP effect estimated by the meta-GWAS is not shown because the linkage phase may be inconsistent among the four populations, and the SNP effect cannot be estimated by the multi-trait GWAS; 5. **: 1% genome-wide significant; *: 5% genome-wide significant; 6. Number of SNPs that surpass the significance level.
