# Supplementary figures and images for: Genome-wide detection of genetic markers associated with growth and fatness in four pig populations using four approaches
Source: Genet Sel Evol. 2017 Feb 14;49:21. doi: 10.1186/s12711-017-0295-4 (PMC5307927; doi:10.1186/s12711-017-0295-4)

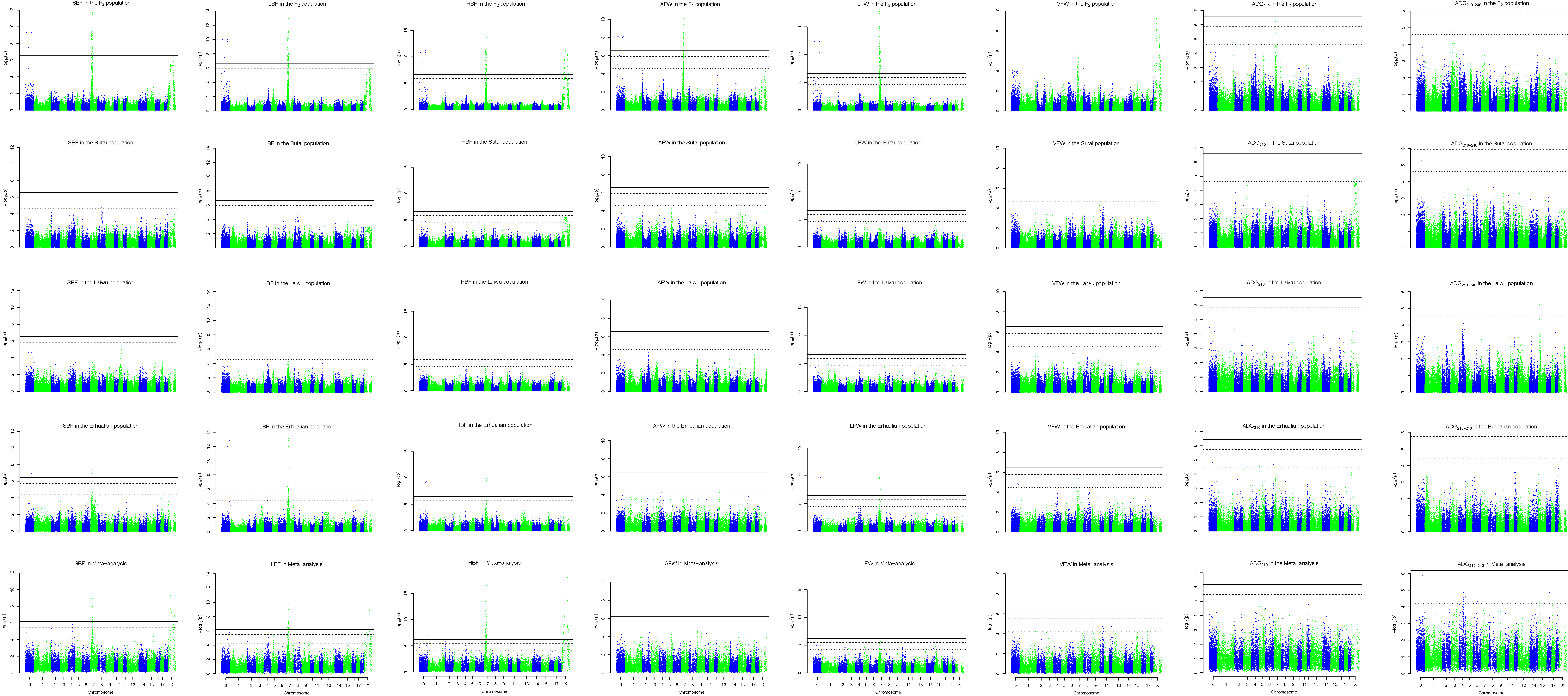

Supplement: Supplementary file 4 — Additional file 4: Fig. S1. Manhattan plots of the single-trait GWAS and meta-analysis for nine fatness and growth traits in the F2, Sutai, Laiwu and Erhualian populations. The solid, dashed and dotted horizontal lines indicate the 1% and 5% genome-wide and suggestive significant threshold values, respectively. Unmapped SNPs are assigned on chromosome 0 and arbitrary ordered by their names. [file 12711_2017_295_MOESM4_ESM.tiff]
