# Supplementary material for: Genome-wide detection of genetic markers associated with growth and fatness in four pig populations using four approaches
Source: Genet Sel Evol. 2017 Feb 14;49:21. doi: 10.1186/s12711-017-0295-4 (PMC5307927; doi:10.1186/s12711-017-0295-4)
Supplement: Supplementary file 5 — Additional file 5: Table S4. The additive effects of suggestive significant SNPs detected by the meta-analysis in the four populations. [file 12711_2017_295_MOESM5_ESM.doc]

**Table S4 The additive effects of suggestive significant SNPs detected by the meta-analysis in the four populations**

| **Chr^1^** | **Trait^2^** | **Top SNP** | **Position, Mb** | **Allele** | **Effect ± SE^3^** | | | |
| --- | --- | --- | --- | --- | --- | --- | --- | --- |
|  |  |  |  |  | **F_2_** | **Sutai** | **Erhualian** | **Laiwu** |
| **1** | HBF | ss107832889 | 279.55 | A | 0.171 ± 0.059^**^ | 0.128 ± 0.045^**^ | -0.045 ± 0.078 | -0.219 ± 0.137 |
| **1** | VFW | ss131154610 | 294.77 | A | -0.046 ± 0.022^*^ | -0.067 ± 0.021^**^ | 0.045 ± 0.028 | -0.078 ± 0.040 |
| **2** | LBF | ss131211507 | 3.61 | A | 0.090 ± 0.037^*^ | 0.088 ± 0.031^**^ | 0.097 ± 0.057 | -0.078 ± 0.091 |
| **2** | FBF | ss131193581 | 9.37 | A | -0.075 ± 0.043 | 0.082 ± 0.037^*^ | -0.009 ± 0.052 | -0.070 ± 0.068 |
| **2** | FBF | ss131193699 | 9.58 | G | -0.062 ± 0.042 | -0.168 ± 0.041^**^ | -0.026 ± 0.080 | 0.075 ± 0.060 |
| **2** | LFW | ss131197985 | 117.07 | C | 0.054 ± 0.069 | -0.101 ± 0.03^**^ | 0.178 ± 0.077^*^ | -0.134 ± 0.092 |
| **2** | AFW | ss131201823 | 132.2 | A | 0.009 ± 0.020 | -0.037 ± 0.013^**^ | 0.032 ± 0.023 | 0.074 ± 0.024^**^ |
| **3** | FBF | ss131215218 | 9.48 | A | 0.054 ± 0.046 | -0.130 ± 0.035^**^ | -0.105 ± 0.057 | -0.090 ± 0.058 |
| **3** | LFW | ss131054825 | 9.96 | G | 0.032 ± 0.042 | 0.112 ± 0.037^**^ | 0.120 ± 0.045^**^ | -0.076 ± 0.070 |
| **3** | AFW | ss107873924 | 109.12 | G | -0.011 ± 0.019 | 0.047 ± 0.014^**^ | -0.048 ± 0.024^*^ | 0.046 ± 0.035 |
| **4** | ADG_0-210_ | ss131267570 | 77.15 | A | -0.017 ± 0.005^**^ | -0.009 ± 0.004^*^ | 0.007 ± 0.005 | 0.008 ± 0.007 |
| **4** | ADG_210-240_ | ss131269053 | 80.96 | A | 0.053 ± 0.021^**^ | -0.024 ± 0.014 | -0.012 ± 0.017 | 0.048 ± 0.013^**^ |
| **4** | LBF | ss131269678 | 82.25 | A | -0.181 ± 0.061^**^ | 0.078 ± 0.035^*^ | 0.071 ± 0.066 | -0.164 ± 0.057^**^ |
| **5** | LFW | ss120032250 | 25.79 | A | 0.089 ± 0.052 | 0.130 ± 0.034^**^ | -0.075 ± 0.055 | -0.145 ± 0.107 |
| **5** | LBF | ss107903259 | 27.07 | A | 0.201 ± 0.053^**^ | 0.088 ± 0.037^*^ | -0.030 ± 0.054 | 0.194 ± 0.073^**^ |
| **5** | ADG_0-210_ | ss478941239 | 28.66 | C | -0.010 ± 0.005^*^ | -0.014 ± 0.005^**^ | 0.008 ± 0.005 | -0.013 ± 0.007 |
| **5** | AFW | ss120032165 | 87.87 | G | -0.021 ± 0.021 | -0.064 ± 0.016^**^ | -0.020 ± 0.030 | -0.075 ± 0.048 |
| **6** | ADG_0-210_ | ss131110597 | 9.27 | A | -0.010 ± 0.004^*^ | -0.012 ± 0.005^**^ | 0.007 ± 0.005 | -0.019 ± 0.010^*^ |
| **6** | ADG_210-240_ | ss131094986 | 90.68 | A | -0.024 ± 0.011^*^ | 0.042 ± 0.013^**^ | -0.015 ± 0.014 | -0.023 ± 0.013 |
| **6** | FBF | ss107829154 | 134.48 | A | 0.189 ± 0.057^**^ | -0.061 ± 0.038 | 0.065 ± 0.074 | -0.174 ± 0.067^**^ |
| **8** | LBF | ss107826859 | 15.01 | A | -0.102 ± 0.072 | -0.094 ± 0.029^**^ | -0.073 ± 0.067 | 0.106 ± 0.052^*^ |
| **8** | AFW | ss131376236 | 117.96 | A | -0.004 ± 0.022 | 0.044 ± 0.010^**^ | 0.015 ± 0.038 | 0.039 ± 0.030 |
| **9** | AFW | ss107867089 | 12.14 | A | 0.013 ± 0.017 | 0.041 ± 0.016^**^ | 0.068 ± 0.041 | 0.089 ± 0.028^**^ |
| **9** | LBF | ss131390688 | 47.49 | G | 0.070 ± 0.050 | 0.107 ± 0.030^**^ | 0.019 ± 0.092 | 0.135 ± 0.082 |
| **10** | LFW | ss131434909 | 12.79 | A | 0.039 ± 0.062 | 0.100 ± 0.027^**^ | 0.102 ± 0.054 | -0.038 ± 0.108 |
| **10** | AFW | ss131032897 | 27.69 | A | 0.017 ± 0.025 | -0.133 ± 0.060^*^ | 0.115 ± 0.031^**^ | 0.041 ± 0.036 |
| **10** | VFW | ss131086122 | 54.23 | A | 0.011 ± 0.019 | 0.064 ± 0.016^**^ | 0.047 ± 0.024^*^ | 0.017 ± 0.034 |
| **12** | ADG_0-210_ | ss131459427 | 31.23 | G | -0.009 ± 0.007 | -0.018 ± 0.007^**^ | 0.012 ± 0.005* | 0.014 ± 0.006* |
| **12** | VFW | ss131073797 | 36.81 | G | 0.032 ± 0.034 | -0.051 ± 0.013^**^ | -0.040 ± 0.044 | 0.055 ± 0.042 |
| **14** | HBF | ss107868905 | 37.75 | A | 0.186 ± 0.072^*^ | 0.114 ± 0.050^*^ | 0.114 ± 0.072 | 0.122 ± 0.072 |
| **14** | LFW | ss131502410 | 137.22 | G | 0.039 ± 0.042 | -0.138 ± 0.041^**^ | -0.056 ± 0.065 | -0.168 ± 0.067^*^ |
| **15** | LFW | ss478935207 | 12.81 | G | 0.037 ± 0.060 | -0.139 ± 0.034^**^ | -0.079 ± 0.064 | 0.084 ± 0.070 |
| **15** | ADG_210-240_ | ss131531233 | 16.45 | G | 0.031 ± 0.011^**^ | -0.016 ± 0.013 | 0.036 ± 0.012^**^ | 0.012 ± 0.011 |
| **15** | FBF | ss120029026 | 121.31 | G | 0.101 ± 0.046^*^ | 0.030 ± 0.064 | -0.176 ± 0.075^*^ | 0.054 ± 0.101 |
| **16** | ADG_210-240_ | ss131536391 | 52.37 | A | 0.046 ± 0.013^**^ | 0.035 ± 0.015^*^ | -0.031 ± 0.015^*^ | -0.008 ± 0.018 |
| **17** | AFW | ss131549596 | 5.76 | G | 0.011 ± 0.019 | 0.047 ± 0.012^**^ | -0.027 ± 0.018 | 0.022 ± 0.024 |
| **17** | VFW | ss131543346 | 19.96 | G | 0.011 ± 0.023 | 0.048 ± 0.013^**^ | -0.025 ± 0.025 | -0.086 ± 0.046 |
| **18** | HBF | ss71868563 | 12.98 | A | 0.095 ± 0.053 | -0.130 ± 0.040^**^ | -0.162 ± 0.064^*^ | 0.055 ± 0.108 |

* 1. Chromosome; 2. The abbreviations of the traits are as same as those in Table S1; 3. **: highly significant (*P* ≤ 0.01); *: significant (*P* ≤ 0.05); without *: non-significant.
