# Supplementary material for: Genome-wide detection of genetic markers associated with growth and fatness in four pig populations using four approaches
Source: Genet Sel Evol. 2017 Feb 14;49:21. doi: 10.1186/s12711-017-0295-4 (PMC5307927; doi:10.1186/s12711-017-0295-4)
Supplement: Supplementary file 6 — Additional file 6: Table S5. Tight linkage of unassigned significant SNPs with the mapped SNPs. This table shows approximate genomic positions of the unassigned significant SNPs by a two-point linkage analysis in the F2 population. [file 12711_2017_295_MOESM6_ESM.doc]

**Table S5** Tight linkage of unassigned significant SNPs with the mapped SNPs

| Unmapped SNP | Population | Associated trait | Tightly linked SNP | | | Recombination | LOD |
| --- | --- | --- | --- | --- | --- | --- | --- |
|  |  |  | Name | Chromosome | Position, bp |  |  |
| ss107885894 | Meta | ADG_0-210_ | ss131070551 | 1 | 106524646 | 0.0000 | 46.06 |
| ss131182077 | Sutai | HBF | - | 2 | 3641788 (3720488) | - | - |
| ss107846837 | Sutai | FBF, LFW | ss131341662 | 2 | 31045816 | 0.0085 | 111.31 |
| ss478937594 | Meta | AFW | ss131226529 | 3 | 114499593 | 0.0040 | 65.20 |
| ss131029816 | Meta | ADG_210-240_ | ss131566312 | 6 | 71595579 | 0.0022 | 400.64 |
| ss131106780 | Erhualian | ADG_0-210_ | ss107897984 | 6 | 157734339 | 0.0000 | 56.59 |
| ss107860263 | Meta | Multi-trait | ss131088233 | 7 | 609952 | 0.0022 | 213.89 |
| ss107860263 | Meta | FBF, LFB, HBF, LFW | ss131088233 | 7 | 609952 | 0.0022 | 213.89 |
| ss107836211 | Erhualian | Multi-trait | ss131341275 | 7 | 29653194 | 0.0040 | 253.26 |
| ss107836211 | Erhualian | SBF, LBF, HBF, LFW | ss131341275 | 7 | 29653194 | 0.0040 | 253.26 |
| ss107879050 | F_2_ | Multi-trait | ss131342502 | 7 | 32997273 | 0.0016 | 404.24 |
| ss107879050 | F_2_ | SBF, FBF, LBF, LFW, AFW | ss131342502 | 7 | 32997273 | 0.0016 | 404.24 |
| ss131101988 | F_2_ | Multi-trait | ss131342502 | 7 | 32997273 | 0.0016 | 404.24 |
| ss131101988 | F_2_ | SBF, FBF, LBF, LFW, AFW | ss131342502 | 7 | 32997273 | 0.0016 | 404.24 |
| ss131342382 | F_2_ | HBF | ss131342502 | 7 | 32997273 | 0.0016 | 404.84 |
| ss131348689* | Meta | SBF | ss107821025 | 7 | 44159396 | 0.0016 | 113.10 |
| ss131031851 | Sutai | ADG_210-240_ | - | 10 | 65380777 (65288213) | - | - |
| ss478942349 | Laiwu | SBF | - | 11 | 44749013 (44835241) | - | - |

* ss131348689 maps to chromosomes 7 and 15 in the SNP database. After two-point linkage analysis, it tightly links to ss107821025 at 42116624 on SSC7.
